# Bee and Beekeeping Research in a Rapidly Changing World: Advancements and Challenges

**DOI:** 10.3390/molecules26113066

**Published:** 2021-05-21

**Authors:** Angelo Canale, Giovanni Benelli

**Affiliations:** Department of Agriculture, Food and Environment, University of Pisa, Via del Borghetto 80, 56124 Pisa, Italy; giovanni.benelli@unipi.it

Populations of pollinating insects are in concrete decline globally [1]. Many wild bees are now considered at risk of extinction, disappearing from many habitats [2]. In the last decade, high attention has been paid to the rarefaction of the populations of *Apis mellifera*, the common honeybee. Populations of this species have suffered significant numerical losses since 2006–2007 in the USA, when the phenomenon of colony collapse disorder (CCD) was first described by B. Oldroyd [3]. The great interest in the decline of this species is justified by the fact that honeybees, in addition to providing important foods and products such as honey, pollen, propolis, royal jelly and venom [4,5,6,7,8], have long been employed for the pollination service [9,10]. Therefore, their decline poses a serious threat to the production of important crops for human consumption globally, with major repercussions on yields.

To face this worrying scenario, we should focus on the complexity of the trophic networks in which the honeybee participates, acting as a reliable indicator of the level of environmental sustainability of a given habitat. Few bees in a specific ecosystem likely indicate an anomaly in progress. However, abundant densities of honeybees, experimentally surveyed in an identified range, are not necessarily an indication of high environmental quality. Bees can be raised in large numbers and transported to a given biotope, thus generating an experimental bias. To overcome this issue, it is necessary to extend the measure not only to honeybees but also to other wild Apoidea (including solitary, gregarious and social species) or, even better, to the cluster of insects present in a specific area. The recent scientific literature focuses the attention on the more generalized decline of insect populations as a whole. Long-term scientific data examining 452 species of invertebrates from 1970s indicate a dramatic population reduction of about 45% [11].

What are the possible causes of this widespread reduction in insects in general and pollinators, more specifically, on a worldwide scale? Current scientific knowledge suggests that a single factor cannot explain the phenomenon of CCD. More generally, the reasons for the rarefaction of the pollinator populations, reared and wild, admit a multifactorial hypothesis, where the causes of perturbation often act concomitantly, giving rise to frequent and amplified synergistic disturbing actions. Important factors responsible for the rarefaction of beneficial insects include—among others—the fragmentation and degradation of habitats, with the consequent loss of natural floristic communities, the models of intensive agricultural management with the excessive use of pesticides, environmental pollution, the increasingly widespread ease of the diffusion of invasive alien species, the pressure of parasites and pathogens, global warming and its associated consequences [1,2,3,12,13].

The case of insecticides belonging to the neonicotinoid class can help one to better understand the complexity of the phenomenon, shedding light on the above-mentioned multifactorial synergistic interactions. Neonicotinoids, a class of molecules obtained synthetically in the mid-90s of the last century, are very similar in structure to nicotine. They have an indirect neurotoxic action, blocking nicotinic receptors and causing nerve over-stimulation and paralysis. They are highly toxic to most arthropods, so they can be used to effectively manage their populations. Being characterized by high mobility at the xylem and phloem level, their use is widespread in modern agriculture. However, the ease with which they are translocated into the vascular tissues of the treated plants often determines significant concentrations of the active principle in the nectar and pollen, thus putting the pollinator entomofauna at risk [12,13]; this is also due to their toxicity through ingestion, which is higher than that achieved by contact. Considering these reasons, for some of the most common active ingredients, the European Food Safety Authority (EFSA) highlighted their danger to honey and wild bees [14]. A number of studies indicate the sub-lethal effects of this class of insecticides, which in the case of honeybees manifest themselves with interactions at the metabolic and neuro-cognitive level, such as the increase in the time of larval development, disturbances in the orientation, reduction in olfactory ability, and learning alteration [15]. For the super-organism *A. mellifera*, the consequences of taking sub-lethal doses of neonicotinoids can be fatal. The symptoms related to the exposure of honeybees to low doses of neonicotinoids highlight side-effects and synergies between stressors, even more subtle than those described above. For instance, sub-lethal doses of clothianidin alter the bee immune response, promoting the replication of the deformed wing virus (DWV) [16]. Colonies exposed to this insecticide are more sensitive and more exposed to attacks by pathogens. Additionally, an unusual mutualistic symbiosis between the DWV and the ectoparasitic bee mite *Varroa destructor*, has been reported. Feeding mites act as DWV vectors in *A. mellifera* colonies. As a counterpart, the DWV induces immuno-suppression in the host, delaying the repair of nutritional wounds inflicted by the *V. destructor* mother, thus favoring the feeding and reproductive success of the mite’s offspring [17]. It is evident how the attempt to solve a problem—i.e., the use of a neonicotinoid to control a phytophagous insect—can trigger a sort of unpredictable domino effect, which is not easy to interrupt. We must acquire greater awareness that whenever we intervene in an ecosystem we alter, in a short time, the consolidated structure and complexity of the trophic networks. Intensive agricultural systems lack the key ecological services needed for the maintenance of their homeostasis. In contrast, a low-input management of agricultural production processes translates, over time, into a better level of biodiversity, generating synergies to support the maintenance of soil fertility, yields and the protection of multifunctional food networks, including tools to support bee populations [18,19]. In our opinion, taking a small step back will allow us to gain the momentum to take a longer leap forward.

In this delicate and timely scenario, *Molecules* welcomes original research articles and reviews on bee research, with special reference to the identification of novel compounds to boost bee health, including products to fight their parasites and pathogens. In addition, as mentioned above, beekeeping is a major source of important products of high value for humans, including honey, pollen, propolis, venom and royal jelly [4,5,6,7,8]. Studies focusing on this topic perfectly fits the aim of *Molecules*, which is committed to publishing beekeeping research and reviews.

As Academic Editors of *Molecules*, in the present article, we selected twelve carefully reviewed articles on bee and beekeeping research published in this international journal [6,20,21,22,23,24,25,26,27,28,29,30]. In the first subset of selected articles, recent advances in the field of pesticide detection in beekeeping products are presented [20,21,22]. A further research field well covered in *Molecules* in recent years is the evaluation of novel natural products to fight honeybee pathogens and parasites [23,24,25]. Finally, major attention has been devoted to the chemical, biological and nutraceutical properties of beekeeping products, with special reference to bee-collected pollen [6,26,27], venom [28], propolis and honey [29,30].

Overall, bee and beekeeping research fits well the aim of *Molecules*, as outlined by the articles mentioned above. On the other hand, we are very aware that this Editorial cannot reflect the many facets and research challenges characterizing this interesting field of research. However, we hope that it can inspire further research in our scientific community, particularly among young researchers.

“*The bee’s life is like a magic well: the more you draw from it, the more it fills with water*”.

Karl Von Frisch [31]
